# Comprehensive Analysis of the Chemical and Bioactivity Profiles of Endemic *Crataegus turcicus* Dönmez in Comparison with Other *Crataegus* Species

**DOI:** 10.3390/molecules28186520

**Published:** 2023-09-08

**Authors:** Tansu Turnalar Ülger, Mehmet Ali Oçkun, Etil Guzelmeric, Nisa Beril Sen, Hande Sipahi, Yağmur Özhan, Yüksel Kan, Erdem Yesilada

**Affiliations:** 1 Department of Pharmacognosy, Graduate School of Health Sciences, Yeditepe University, Kayisdagi Cad., Atasehir, 34755 Istanbul, Turkey; tansu.turnalar@gmail.com; 2 Faculty of Pharmacy, Yeditepe University, Kayisdagi Cad., Atasehir, 34755 Istanbul, Turkey; mehmet.ockun@yeditepe.edu.tr; 3 Department of Pharmacognosy, Faculty of Pharmacy, Yeditepe University, Kayisdagi Cad., Atasehir, 34755 Istanbul, Turkey; nisaberil.sen@yeditepe.edu.tr; 4 Department of Toxicology, Faculty of Pharmacy, Yeditepe University, Kayisdagi Cad., Atasehir, 34755 Istanbul, Turkey; hande.sipahi@yeditepe.edu.tr (H.S.); yagmur.ozhan@yeditepe.edu.tr (Y.Ö.); 5 Department of Medicinal Plants, Agriculture Faculty, Selçuk University, 42130 Konya, Turkey; ykan@selcuk.edu.tr

**Keywords:** *Crataegus* L., *Crataegus turcicus*, phenolic profiling, antioxidant activity, anti-inflammatory activity, analgesic activity

## Abstract

*Crataegus turcicus* is a plant endemic to Türkiye. For the first time, this study aimed to comparatively assess its flower-bearing branches, leaves, and fruits with other well-known *Crataegus* species (*C. monogyna*, *C. pentagyna*, and *C. orientalis*) in terms of chemical composition and bioactivity studies to evaluate its potential use as a food supplement. Firstly, the contents of total phenolics (TPC), flavonoids (TFC), proanthocyanidin (TPAC), and anthocyanin (TAC) in different plant parts of *Crataegus* species were evaluated. The highest TPAC was found in the hydroalcoholic extract of *C. turcicus* flower-bearing branches. Moreover, all plant parts had comparatively higher amounts of TPC, TFC, and TAC compared to other *Crataegus* species. The chemical screening by high-performance thin-layer chromatography (HPTLC) resulted that *C. turcicus* parts were rich with chlorogenic acid, neochlorogenic acid, quercetin and vitexin derivatives, epicatechin, procyanidin, etc., and their quantities were evaluated by high-performance liquid chromatography (HPLC). In terms of several *in vitro* antioxidant activity outcomes, the flower-bearing branches of *C. turcicus* showed the highest antioxidant activity by a 2,2-diphenyl-1-picrylhydrazyl (DPPH) test among the assessed antioxidant assays. Additionally, hydroalcoholic extracts of *C. turcicus* significantly decreased LPS-induced nitric oxide, tumor necrosis factor-alpha, and interleukin-6 production more potently than indomethacin (positive control). In addition to its remarkable anti-inflammatory activity, *C. turcicus* showed analgesic activity by reducing prostaglandin E_2_ levels.

## 1. Introduction

Reactive oxygen species (ROS), such as superoxide radicals, hydrogen peroxide, and hydroxyl radicals are produced as by-products of cellular metabolism. If the defense mechanism may not be able to detoxify these reactive species by converting them into nonradical metabolites, oxidative stress may occur, and it may lead to occurrence of chronic inflammation [1]. Basically, production of ROS and eliminating mechanisms should be in balance for preventing diseases. Bioactive natural compounds, especially phenolics, may serve as a preventive treatment because of their antioxidant and anti-inflammatory activities [2,3].

**Figure 1:**
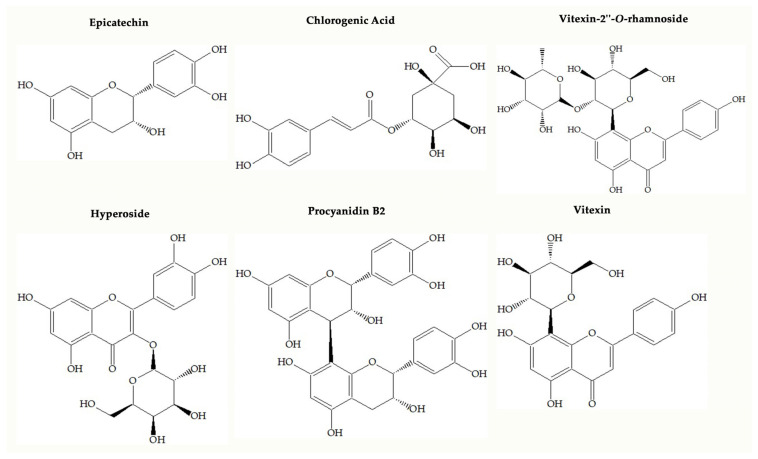
Chemical structures of main compounds found in *Crataegus* species.

One of the popular food supplements that is formulated based on *Crataegus* spp. (Hawthorn) may have an important role in preventing oxidative stress because it has a wide range of phenolic compounds. Up to now, scientific papers have shown that *Crataegus* species may bear flavonoids (hyperoside, rutin, isoquercitrin, vitexin, vitexin-2″-*O*-rhamnoside, orientin, isoorientin, etc.), oligomeric procyanidins (procyanidins B2 and B5, epicatechin, etc.) and some phenolic acids such as chlorogenic acid, neochlorogenic acid, protocatechuic acid, and caffeic acid, illustrated in Figure 1 [4,5,6]. Oligomeric procyanidins are abundant in fruits, whereas flavonols such as flavonol glycosides and C-glycosyl flavones are dominant in leaves and flowers of the *Crataegus* species [4].

Traditionally, different plant parts of *Crataegus* species prepared mostly by infusion and decoction techniques have been used for the treatment of cardiovascular diseases such as hypertension, palpitations, and tachycardia. In addition, it is used for the treatment of respiratory tract problems such as bronchitis and asthma, diarrhea, and abdominal pains [7,8]. In addition, C-glycosyl flavones in the structure of *Crataegus* species affect inflammation by limiting the formation of cytokines, nitric oxide, and prostaglandin E2 in tissues and increasing the level of anti-inflammatory cytokines [9]. Also, research on different plant parts of *Crataegus* species clarified that it has other biological effects such as antioxidant, antimicrobial, gastroprotective, cardioprotective, anti-anxiety, anti-depression, cardiotonic, anti-atherogenic, hypoglycemic, and diuretic effects [10,11,12,13,14,15,16,17,18]. *Crataegus* species are described as “Generally recognized as safe, GRAS” and registered as “Traditional herbal medicinal product” in the Committee for Herbal Medicinal Products of the European Medicines Agency (EMA, 2016) [19]. In many pharmacopeias as European Pharmacopoeia, either fruits or flower-bearing branches of *Crataegus monogyna* and *C. laevigata* (syn. *C. oxyacantha*), flower-bearing branches of *C. pentagyna*, *C. nigra*, and *C. azarolus* are registered as an official plant material for medical usage [20].

A recent review on *Crataegus* L. has declared that the chemistry of at least 27 *Crataegus* species has been thoroughly described so far [8]. On the other hand, there are limited data available either on chemical composition or bioactivity profiles on *Crataegus turcicus* Dönmez, which is endemic to Türkiye. *C. turcicus* was first described in the Ardanuç (Artvin) district (Türkiye) by Dönmez and Dönmez [21]. *C. turcicus* is a small tree or shrub about up to 6 m in height. The branches are thornless or rarely have thorns 1.5–3 cm long. The upper surface of the subterminal leaves on flowering branches is green, the lower surface is grayish-green, 35–55 × 35–60 mm, and the margin is irregularly serrated. Its sepals are 2–3.5 × 1–2 mm, its petals are 4–6 × 4–5 mm, stamens are 20, and its styles are 4–5. Its false fruits are dark red, and its pyrene is 7–9 × 4–4.5 mm and 4–5 [21].

This study aimed to comparatively evaluate chemical compositions and bioactivities of endemic *C. turcicus* flower-bearing branches, leaves, and fruits with very well-known *Crataegus* species as *C. monogyna*, *C. pentagyna*, and *C. orientalis*. Within this study, total phenolic (TPC), flavonoid (TFC), proanthocyanidin (TPAC), and anthocyanin (TAC) contents of different plant parts of *Crataegus* species were evaluated by *in vitro* tests. Then, high-performance thin-layer chromatography (HPTLC) was used for the chemical profiling of the compounds in *Crataegus* hydroalcoholic extracts belonging to flower-bearing branches, leaves, and fruits. The main compounds were quantified with high-performance liquid chromatography (HPLC). Hydroalcoholic extracts of *Crataegus* species were further subjected to *in vitro* tests to comparatively evaluate their antioxidant, anti-inflammatory, and analgesic activities.

This is the first study comparatively evaluating potential usage of *C. turcicus* as a food supplement with registered *Crataegus* species in pharmacopoeias as *C. monogyna* and *C. pentagyna* as well as traditionally widely used *C. orientalis*.

## 2. Results

### 2.1. Results of Total Phenolic (TPCs), Flavonoid (TFCs), Proanthocyanidin (TPACs), and Anthocyanin (TACs) Contents

TPCs of hydroalcoholic extracts of flower-bearing branches, leaves, and fruits belonging to different *Crataegus* species were demonstrated in Table 1. Among the hydroalcoholic extracts of flower-bearing branches, *C. pentagyna* (≈369 mg GAE/g) had the highest content of TPC, followed by *C. monogyna* (≈302 mg GAE/g) and *C. orientalis* (≈288 mg GAE/g). *C. orientalis* leaves showed the highest TPC (≈874 mg GAE/g). Among fruits, the statistically highest TPC was detected in *C. monogyna* (≈116 mg GAE/g) and *C. pentagyna* (≈91 mg GAE/g). All of the fruits belonging to different *Crataegus* species had the lowest TPC than the other parts. The results of the TFCs of *Crataegus* species are shown in Table 1. Considering the flower-bearing branches, *C. pentagyna* (≈27 mg QE/g) showed the highest TFC. *C. pentagyna* (≈64 mg QE/g) leaves demonstrated higher TFC results than the plant parts of other *Crataegus* species. The second statistically higher results were observed in *C. turcicus* (≈34 mg QE/g) and *C. orientalis* (≈38 mg QE/g) leaves. In the perspective of fruits, the *Crataegus* species had the lowest TFC. Based on the outcomes of the TPAC, the flower-bearing branches of *C. turcicus, C. monogyna,* and *C. orientalis* exerted statistically the highest TPACs, as follows: ≈31 mg CCE/g, ≈30 mg CCE/g, and ≈29 mg CCE/g, respectively. According to the results of TAC, shown in Table 1, *C. pentagyna* (≈15 mg C3GE/g) demonstrated the highest TAC, followed by *C. turcicus* (≈6 mg C3GE/g).

### 2.2. HPTLC

**Figure 2:**
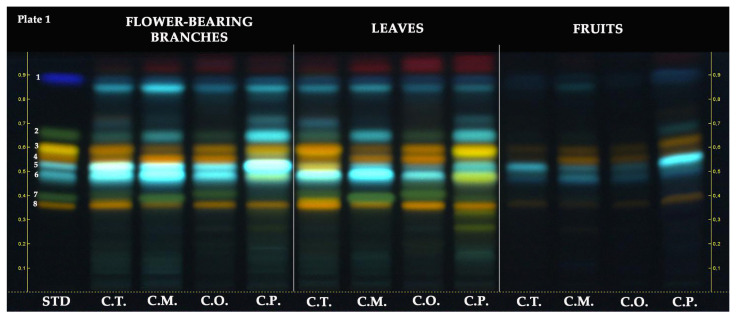
HPTLC chromatogram of different parts of *Crataegus* species at 366 nm. STD: standards, 1: protocatechuic acid, 2: vitexin, 3: isoquercitrin and orientin, 4: hyperoside, 5: neochlorogenic acid, 6: chlorogenic acid, 7: vitexin-2″-*O*-rhamnoside, and 8: rutin.

The HPTLC chromatogram of the hydroalcoholic extracts revealed the following retention factors (R_F_) for the standards: rutin (R_F_ ≈ 0.36), vitexin-2″-*O*-rhamnoside (R*_F_* ≈ 0.40), chlorogenic acid (R_F_ ≈ 0.48), neochlorogenic acid (R_F_ ≈ 0.53), hyperoside (R_F_ ≈ 0.56), isoquercitrin (R_F_ ≈ 0.59), orientin (R_F_ ≈ 0.59), vitexin (R_F_ ≈ 0.66), and protocatechuic acid (R_F_ ≈ 0.88). As depicted in Figure 2, rutin (R_F_ ≈ 0.36, orange-colored zone) was observed in all *Crataegus* samples. Rutin was commonly observed in samples of flower-bearing branches and leaves compared to fruit samples. Among the flower-bearing branches, notable presences of rutin were found in *C. turcicus, C. orientalis,* and *C. pentagyna.* In leaves, rutin was mainly observed in *C. turcicus, C. orientalis,* and *C.pengayna.* Vitexin-2″-*O*-rhamnoside (R_F_ ≈ 0.40, green-colored zone) was observed in all samples of the *Crataegus* species except for fruit samples, and it was predominantly observed in *C. monogyna* flower-bearing branches and leaves and *C. orientalis* leaves. While chlorogenic acid (R_F_ ≈ 0.48, light blue-colored zone) was detected in all *Crataegus* samples, it was mainly found in *C. turcicus* and *C. monoygna* flower-bearing branches and *C. turcicus* and *C. monoygna* leaves. Neochlorogenic acid (R_F_ ≈ 0.53, light blue-colored zone) was detected in all *Crataegus* samples except *C. turcicus* leaves. It was mainly found in the flower-bearing branches of *C. turcicus, C. monogyna*, and *C. pentagyna*. Among the fruits, it was notably observed in *C. pentagyna.* Hyperoside (R_F_ ≈ 0.56, orange-colored zone) was slightly observed in all fruit samples compared to flower-bearing branches and leaves of *Crataegus* species. It was notably found in *C. monogyna* flower-bearing branches and *C. orientalis* leaves. Isoquercitrin and orientin had the same R_F_ value due to co-migrated on the stationary phase. Orientin (R_F_ ≈ 0.59, yellow-colored zone) was predominantly detected in *C. pentagyna* leaves. In addition, it was also found in *C. pentagyna* flower-bearing branches and *C. turcicus* leaves. Isoquercitrin (R_F_ ≈ 0.59, orange-colored zone) was detected in all samples, whereas it was notably observed in flower-bearing branches and leaves of *Crataegus* species. The green band color belonging to vitexin at R_F_ ≈ 0.66 was not clearly observed on the HPTLC plate. This could have been due to the blue-colored compound at the same R_F_ value with vitexin, potentially a phenolic acid, co-migrated with it. All *Crataegus* species may possibly have a protocatechuic acid (R_F_ ≈ 0.88, dark blue-colored zone).

**Figure 3:**
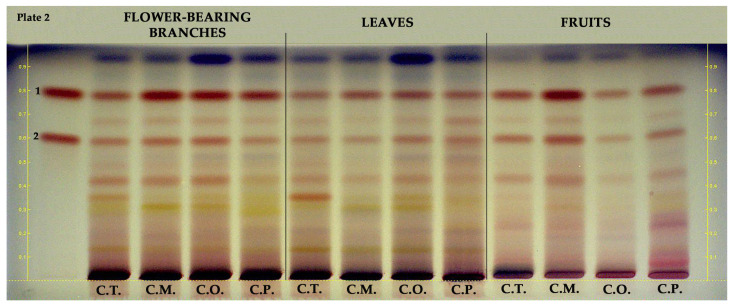
HPTLC chromatogram of different parts of *Crataegus* species at white light. 1: epicatechin, 2: procyanidin B2, C.T.: *C. turcicus*, C.M.: *C. monogyna*, C.O.: *C. orientalis*, and C.P.: *C. pentagyna*.

Epicatechin and procyanidin B2 in hydroalcoholic extracts of flower-bearing branches, leaves, and fruits of *Crataegus* species were investigated using HPTLC. As shown in Figure 3, epicatechin (R_F_ ≈ 0.8, red band color) was found in all samples and mainly detected in flower-bearing branches of *C. orientalis*, *C. pentagyna,* and *C. monogyna* as well as *C. monogyna* fruits. Procyanidin B2 (R_F_ ≈ 0.6, red band color) was found in all samples and notably detected in *C. monogyna* fruits.

**Figure 4:**
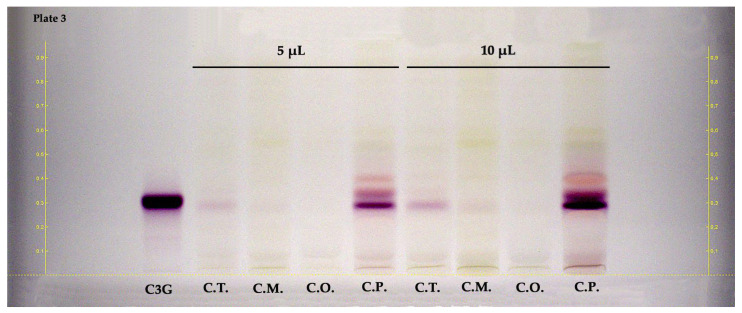
HPTLC chromatogram of fruits belong to *Crataegus* species at white light. C3G: cyanidin 3-*O*-glucoside, C.T.: *C. turcicus*, C.M.: *C. monogyna*, C.O.: *C. orientalis*, and C.P.: *C. pentagyna*.

HPTLC was also applied to examine anthocyanins in the hydroalcoholic extracts of fruit parts of the *Crataegus* species. Figure 4 illustrates the HPTLC chromatogram of the examined samples. The R_F_ values and band color of the standards were provided at white light without undergoing a derivatization process. According to the results of the HPTLC plate (shown in Figure 4), cyanidin-3-*O*-glucoside (R_F_ ≈ 0.31, reddish-colored zone) was dominantly detected in the fruits of *C. pentagyna.* Other reddish-colored zones may confirm the presence of other anthocyanins in the fruits.

### 2.3. HPLC

**Figure 5:**
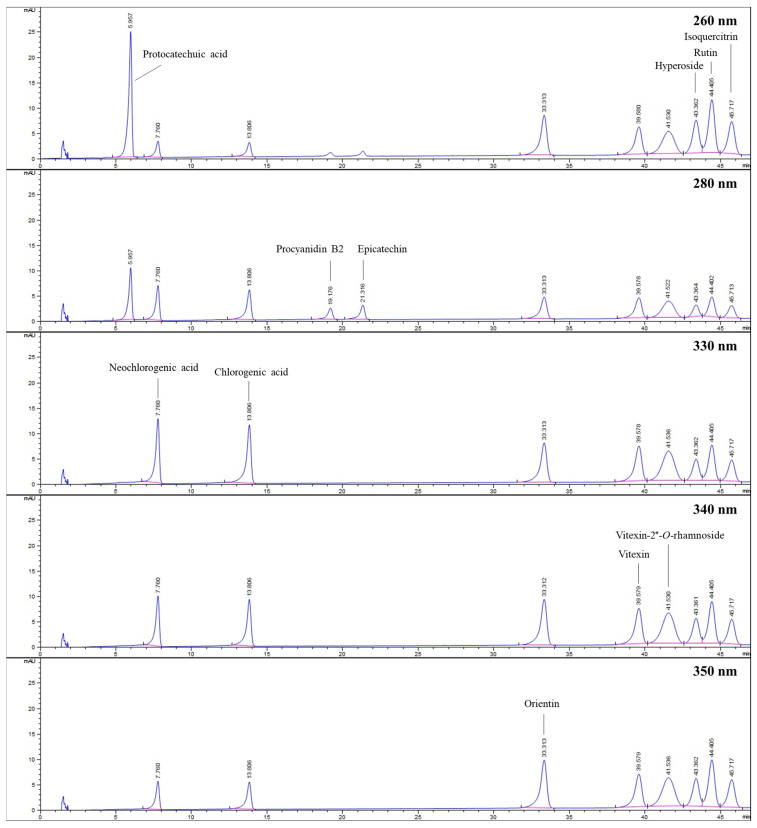
HPLC chromatograms belong to standards investigated at different wavelengths.

The HPLC method was used to determine the amounts of various compounds, including protocatechuic acid, chlorogenic acid, neochlorogenic acid, procyanidin B2, epicatechin, orientin, vitexin, vitexin-2″-*O*-rhamnoside, hyperoside, rutin, and isoquercitrin, in hydroalcoholic extracts of flower-bearing branches, leaves, and fruits belong to *C. turcicus, C. monogyna, C. orientalis*, and *C. pentagyna*. Figure 5 displays the HPLC chromatogram of the standard mixture indicating their retention time (*t*_R_) values.

Table 2 presents the found amounts of the investigated compounds in the *Crataegus* species. According to these results, low amounts of protocatechuic acid were calculated in all samples, its presence was not even detected in fruits of *C. monogyna* and *C. orientalis*. Neochlorogenic acid was present in all samples except for *C. turcicus* leaves. It was predominantly found in *C. pentagyna* flower-bearing branches (≈50 mg/g). Chlorogenic acid, which was detected in all samples, was found as the dominant compound in *C. monogyna* flower-bearing branches (≈46 mg/g). The highest content of procyanidin B2 was calculated in *C. monogyna* fruits (≈12 mg/g), whereas its lowest content was found in *C. turcucis* leaves (≈2 mg/g). The flower-bearing branches of *C. monogyna* had the highest quantities of epicatechin (≈18 mg/g) and hyperoside (≈17 mg/g). The highest amounts of orientin (≈14 mg/g) and vitexin (≈5 mg/g) were detected in hydroalcoholic extracts of *C. pentagyna* leaves. The highest amount for rutin belonged to hydroalcoholic extracts of *C. orientalis* and *C. pentagyna* leaves in the amount of approximately 6 mg/g. In addition, hydroalcoholic extracts of *C. pentagyna* leaves had the highest amount of isoquercitrin (≈13 mg/g). In samples belonging to *C. turcicus*, the peak of isoquercitrin overlapped with another co-eluted compound, so its quantity could not be determined.

### 2.4. Bioactivity Assays

#### 2.4.1. Total Antioxidant Content

According to the DPPH (2,2-diphenyl-1-picrylhydrazyl), the radical scavenging activity results of the hydroalcoholic extract of flower-bearing branches belonging to *C. turcicus* demonstrated the highest antioxidant activities (≈467 mg TE/g). Among the leaves of *Crataegus* species, *C. turcicus* (≈347 mg TE/g) showed the highest radical scavenging activities. While the fruits of *C. monogyna* (≈133 mg TE/g), *C. pentagyna* (≈160 mg TE/g), and *C. turcicus* (≈130 mg TE/g) possessed statistically similar results, the fruits of *C. orientalis* (≈70 mg TE/g) showed the lowest antioxidant capacity (Table 3). According to ABTS (2,2′-azino-bis(3-ethylbenzothiazoline-6-sulfonic acid diammonium salt) radical scavenging activities, *C. monogyna* flower-bearing branches (≈457 mg TE/g) showed the highest antioxidant activities, followed by flower-bearing branches of *C. pentagyna* (≈443 mg TE/g) and *C. orientalis* (≈400 mg TE/g). Among hydroalcoholic extracts of fruits, *C. monogyna* (≈119 mg TE/g) had significant differences and demonstrated the highest activities (Table 3). The ferric-reducing antioxidant potential (FRAP) of *C. monogyna* flower-bearing branches possessed the highest value (≈373 mg TE/g), followed by flower-bearing *C. turcicus* (≈304 mg TE/g). Considering the leaves of four types of *Crataegus* species, *C. turcicus* (≈227 mg TE/g) demonstrated the highest antioxidant activities (Table 3). As presented in Table 3, cupric-reducing antioxidant activities (CUPRACs) of flower-bearing branches, leaves, and fruits of *Crataegus* species and flower-bearing branches of *Crataegus* species showed the highest activities, followed by leaves and fruits, respectively. Among all samples, *C. monogyna* flower-bearing branches (≈852 mg TE/g) had the highest antioxidant activities, whereas *C. pentagyna* fruits (≈181 mg TE/g) possessed the lowest activities. Among the investigated hydroalcoholic extracts of leaves belong to *Crataegus* species, antioxidant activity outcomes showed that *C. turcicus* leaves had the highest antioxidant potential (≈693 mg TE/g).

#### 2.4.2. Anti-Inflammatory Activity

##### Effects of *Crataegus* Extracts on the Cell Viability of RAW 264.7 Macrophages

As an initial step, non-toxic concentrations of extracts with a cell viability of more than 70% were determined before evaluating anti-inflammatory and analgesic activities. The MTT assay showed that all extracts had no cytotoxic effects at concentrations up to 1 mg/mL at 24 h as compared with control cells (received no treatment), shown in Table 4.

##### Nitric Oxide Production in LPS-Stimulated RAW 264.7 Cells

**Figure 6:**
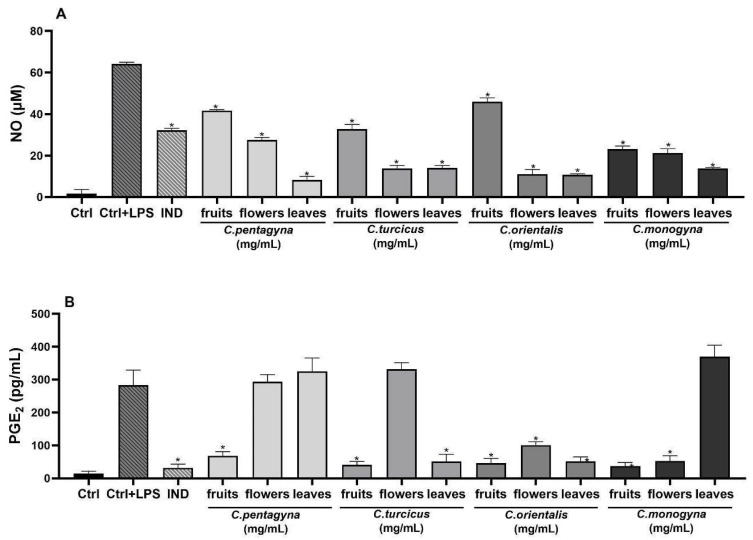

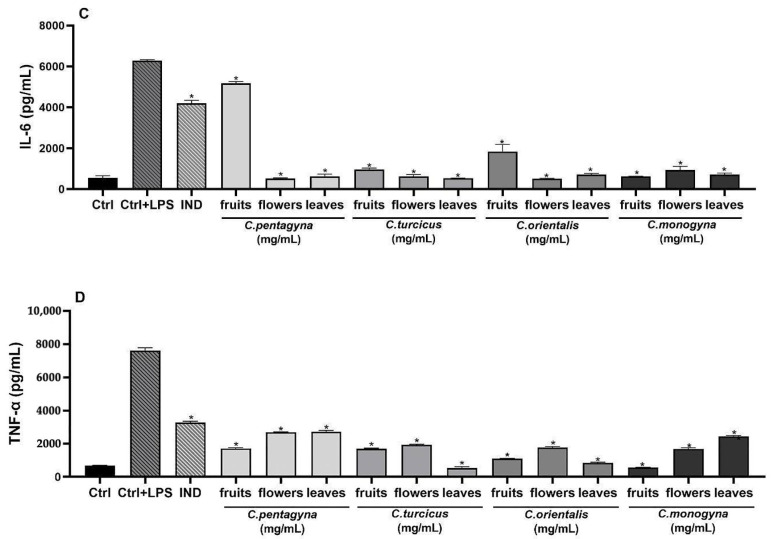
The effects of extracts at 1 mg/mL concentration on nitrite production (**A**), PGE_2_ (**B**), IL-6 (**C**), and TNF-α (**D**) production on LPS stimulated RAW 264.7 cell. Ctrl: Control group treated with DMEM; Ctrl + LPS: Control group only stimulated with LPS; LPS: Lipopolysaccharides from *E. coli*; and IND: Indomethacin (100 µM). Statistical significant differences were indicated for each compound vs. LPS (* *p* < 0.0001).

This study aimed to assess the capabilities of hydroalcoholic extracts of *Crataegus* species to hinder the production of nitrite induced by LPS in RAW 264.7 cells. In this investigation, indomethacin (positive control) was employed as a well-known anti-inflammatory agent. Treatment with LPS (1 µg/mL) led to a significant increase in nitrite levels in the cell culture supernatant (Table 4). NO production after LPS stimulation increased to 64.17 ± 0.84 μM in the untreated cells. However, all of the extracts exhibited a concentration-dependent reduction in LPS-induced nitrite production, as shown in Table 4. Comparison of anti-inflammatory activities of the hydroalcoholic extracts with the reference molecule, indomethacin (100 µM), are also shown in Table 4. The treatment of indomethacin (100 µM) significantly inhibited nitrite oxide production 50% (*p* < 0.0001). Specifically, the anti-inflammatory efficacy of *C. pentagyna* leaves was notably elevated at the highest concentration (1 mg/mL) when tested on RAW 264.7 cells stimulated with LPS, demonstrating a significant difference (*p* < 0.0001) in comparison to the control group. Moreover, nitrite inhibition of *C. orientalis* leaves and *C. orientalis* flower-bearing branches at their highest studied concentrations was 83%. As a result, all extracts at 1 mg/mL concentration have shown a remarkable anti-inflammatory activity in comparison to LPS control (Figure 6A).

##### Evaluation of Analgesic Activity (PGE_2_ Assay)

The analgesic activity on PGE_2_ productions was evaluated for the highest non-toxic concentration of *Crataegus* hydroalcoholic extracts showing the highest anti-inflammatory activities (1 mg/mL). As shown in Figure 6B, PGE_2_ production was remarkably induced in LPS-stimulated RAW 264.7 macrophages when compared with unstimulated negative controls. According to the results of PGE_2_ level, the hydroalcoholic extract of *C. monogyna* fruit showed the most remarkable decline in PGE_2_ level (37.1 ± 10.81 pg/mL) among the other *Crataegus* species (*p* < 0.0001). However, experimental positive control indomethacin (31.65 ± 11.42 pg/mL) exhibited the most potent PGE_2_ inhibition compared to the LPS-activated control group (282.85 ± 46.13 pg/mL) (Figure 6B).

##### Interleukin (IL)-6-Releasing Inhibition Assay

Activation of inflammation in RAW 264.7 cells was triggered upon exposure to LPS, evident through a notable increase in IL-6 secretion within the supernatant following 24 h of LPS stimulation. However, pretreatment with *Crataegus* hydroalcoholic extracts at a concentration of 1 mg/mL prior to LPS exposure effectively suppressed the heightened secretion of this cytokine. The IL-6 production was more pronounced in the group treated with LPS compared to the control group. As shown in Figure 6C, all compounds suppressed the levels of IL-6 at 1 mg/mL concentrations when compared to the LPS treated group. According to this findings, LPS-activated release of IL-6 significantly declined with a 1 mg/mL concentration of *C. orientalis* flower-bearing branches pre-treatment, whereas *C. pentagyna* was the second most active extract compared to control (515.77 ± 20.63 pg/mL; 525.43 ± 23.06 pg/mL, respectively) (*p* < 0.0001).

##### Tumor Necrosis Factor alpha (TNF-α)

The pro-inflammatory cytokine TNF-α, generated by macrophages and monocytes in response to acute inflammation, plays a pivotal role in triggering various intracellular signaling processes that lead to necrosis or apoptosis [22]. Stimulation with LPS notably increased the levels of the pro-inflammatory cytokine TNF-α (as shown in Figure 6D). In contrast, treatment with *Crataegus* species at high concentrations (1 mg/mL) significantly inhibited the levels of TNF-α that were induced by LPS. *C. turcicus* leaves, *C. monogyna* fruits, and *C. orientalis* leaves reduced LPS-stimulated TNF-α production significantly (93%, 93%, and 89%, respectively). These extracts reduced TNF-α levels almost to medium control levels. Moreover, these extracts were more potent than the indomethacin (57%) used as a positive control. 

## 3. Discussion

In a study conducted by Bardakçı et al. [23], the extracts of *C. turcicus* fruits was compared to the extracts of *C. monogyna* and *C. orientalis* fruits in terms of total phenolic content (TPC) values. In contrast to the present study, their results given as gallic acid equivalent (GAE) per g showed that the extracts of *C. turcicus* (≈398 mg GAE/g) and *C. monogyna* (≈392 mg GAE/g) fruits had statistically equal TPC values and these values were higher than the *C. orientalis* fruit extract (≈381 mg GAE/g). The difference in results could be attributed to the plant collection time and variations in the extraction methods used in the experiments. In another study, Catrinel et al. [24] found the TPC content of a *C. pentagyna* leaves extract, which was ≈207 mg GAE/g higher than the flower extract (≈185 mg GAE/g). Similarly, in the same study, the *C. pentagyna* leaves extract had a higher TPC content compared to its flower-bearing branches. Due to the lack of studies on *C. turcicus*, flower-bearing branches and leaves of the plant could not be compared with other research findings in terms of TPC.

Bardakçı et al. [23] stated the total flavonoid content (TFC) of a *C. turcicus* fruit extract was a ≈24 mg quercetin equivalent (QE) per g extract, which was higher than *C. orientalis* (≈20 mg QE/g) and *C. monogyna* (≈18 mg QE/g) fruit extracts. Barros et al. [25] reported that the TFC value of petroleum ether extract of *C. monogyna* flowers was ≈104 mg catechin equivalents/g, and this value was higher than the fruit part, which was found as 22 mg/g. In addition, the authors noted extracts of both flower and flower-bearing branches belonging to *C. monogyna* had higher TFCs than the fruit part. This result was found to be similar to obtained results in this study.

According to the evaluated total proanthocyanidin content (TPAC), the flower-bearing branches of *Crataegus* species yielded the highest results, followed by leaves and fruits, respectively. Similarly, in a study conducted by Catrinel et al. [24], the TPAC value of *C. pentagyna* flower extract (≈98 mg cyanidin/g) was higher than *C. pentagyna* leaves (≈69 mg cyanidin/g). Froehlicher et al. [26] stated that a *C. monogyna* flower extract, which was ≈6 mg cyanidin/g, had significantly higher TPAC than a fruit (≈1 mg cyanidin/g) extract. Bardakçı et al. [23] conducted a study on the fruits of *C. turcicus*, *C. monogyna*, and *C. orientalis* and found that TPAC values were correlated with the current study. They found that *C. monogyna* had the highest TPAC value, followed by *C. turcicus* and *C. orientalis;* these results were similar to the obtained results in this study. Due to the scarcity of available research on *C. turcicus*, it was not possible to compare the leaves and hydroalcoholic extracts of flower-bearing branches of this plant with the findings of other studies in terms of TPAC. 

In this study, *C. pentagyna* fruit extracts with ≈15 mg cyanidin-3-*O*-glucoside equivalent (C3GE) per g fruit extract had significantly higher total anthocyanin content (TAC) than the other investigated species. In a study conducted by Froehlicher et al. [26], the TAC value was reported as ≈0.2 mg C3GE/g dry weight in the 80% hydroalcoholic extract of *C. monogyna* fruits, whereas the TAC content of *C. monogyna* fruits prepared with 75% hydroalcoholic extract was found as 0.03 mg malvinidin-3-glucoside equivalent/g extract in another study by Stanković et al. [27]. There is no study available related to TAC values of *C. turcicus*, *C. orientalis*, and *C. pentagyna* fruits.

The phenolic profiles of the *Crataegus* species were investigated in detail by using HPTLC. In terms of investigated compounds, *C. turcicus* showed similar HPTLC fingerprinting with that of other *Crataegus* species. The chemical composition is directly linked with the pharmacological activity of a plant extract. Therefore, it can be assumed that *C. turcicus* may have a similar pharmaceutical activity potential with that of officinal species. Bardakçı et al. [23] conducted qualitative and quantitative HPTLC analysis to investigate hydroalcoholic fruit extracts belonging to *C. monogyna*, *C. orientalis*, *C. pontica*, *C. rhipidophylla*, and *C. turcicus* in terms of hyperoside and chlorogenic acid. These compounds were found to be common in all samples. Similarly, in this study hyperoside and chlorogenic acid were detected in all plant parts of *Crataegus* species except *C. pentagyna* leaves which hyperoside could not be found. Bubueanu et al. [28] also performed HPTLC analysis to determine the presence of hyperoside, chlorogenic acid, and rutin in the hydroalcoholic extracts of *C. monogyna* leaves and flowers. They showed that only hyperoside was detected in all samples. On the other hand, in the current study, hydroalcoholic extracts of *C. monogyna* leaves and flower-bearing branches were found to contain hyperoside, chlorogenic acid, and rutin. In addition, Khokhlova et al. [29] analyzed methanolic fruit extracts of different *Crataegus* species by HPTLC. As in the present study, they detected rutin, hyperoside, and chlorogenic acid both in *C. monogyna* and *C. pentagyna.*

In the current study, HPLC analysis was performed to quantify the compounds (protocatachuic acid, neochlorogenic acid, chlorogenic acid, procyanidin B2, epicatechin, orientin, vitexin, vitexin-2″-*O*-rhamnoside, hyperoside, rutin, and isoquercitrin) present in the hydroalcoholic extracts of flower-bearing branches, leaves, and fruits of *C. turcicus*, *C. monogyna*, *C. orientalis*, and *C. pentagyna*. Alirezalu et al. [30] conducted a study on the hydroalcoholic extracts of fruits of 15 different *Crataegus* species, in which they quantitatively analyzed the chlorogenic acid, vitexin, vitexin-2″-*O*-rhamnoside, rutin, hyperoside, and isoquercitrin contents using HPLC. The results revealed that *C. orientalis* had the highest content of chlorogenic acid (≈1 mg/g), as well as it was followed by the *C. pentagyna* (≈0.50 mg/g) and *C. monogyna* (≈0.40 mg/g). Vitexin-2″-*O*-rhamnoside was not detected in the extracts, whereas vitexin was found only in fruits of *C. monogyna* (≈0.2 mg/g) and *C. pentagyna* (≈0.1 mg/g). Rutin was absent in *C. monogyna,* but a higher quantity was found in *C. pentagyna* (≈1.3 mg/g) than in *C. orientalis* (≈0.4 mg/g). *C. pentagyna* also exhibited the highest hyperoside (≈2.4 mg/g) and isoquercitrin (≈1.2 mg/g) contents, followed by *C. orientalis* (≈1.6 mg/g for hyperoside; ≈0.8 mg/g for isoquercitrin) and *C. monogyna* (≈0.2 mg/g for hyperoside, ≈0.7 mg/g for isoquercitrin). Stoenescu et al. [31] also comparatively analyzed the methanolic extracts of fruits, leaves, and flowers of *C. monogyna* and *C. pentagyna* in terms of their phenolic contents (chlorogenic acid, neochlorogenic acid, epicatechin, and rutin) by using HPLC. According to their results, epicatechin was not detected in the leaves and flowers of *C. monogyna* and *C. pentagyna*, whereas it was found in significantly higher amounts in the fruit extracts of *C. monogyna* (≈1 mg/g) than *C. pentagyna* (≈0.4 mg/g). Like the current study, rutin was detected in the highest amounts in the leaves of *C. monogyna* (≈2 mg/g) and *C. pentagyna* (≈2 mg/g), followed by the flower and fruit extracts, respectively. Chlorogenic acid was found in the highest amounts in the leaves extract of *C. monogyna* (≈5 mg/g), whereas the flower extract of *C. pentagyna* had the highest quantity of neochlorogenic acid (≈6 mg/g). In the study conducted by Pavlovic et al. [32], they investigated different plant parts of *C. pentagyna* in different harvesting seasons in May (flowers and leaves) and September (fruits). Accordingly, 80% acetone extracts of the fruits, leaves, and flowers of *C. pentagyna*, phenolic components (neochlorogenic acid, chlorogenic acid, rutin, hyperoside, isoquercitrin, procyanidin B2, and epicatechin) were quantified by using HPLC. According to the obtained results, epicatechin (≈13 mg/g) and procyanidin B2 (≈11 mg/g) were found to be the most abundant compound in the fruits extract. Among the phenolic acids, despite the high amount of chlorogenic acid in leaves (≈5 mg/g), neochlorogenic acid was found to be highest in the flowers extract (≈5 mg/g), supporting the results belonging to this study. In addition, rutin (≈1 mg/g) and hyperoside (≈5 mg/g) were found more abundant in the leaves extract, whereas isoquercitrin (≈3 mg/g) was detected in higher quantities in the flowers extract of *C. pentagyna*. According to the results in this study, hyperoside was not detected in the *C. pentagyna* leaves extract, contrary to the previous study. Luis et al. [33] performed an HPLC study using the methanolic extracts of the leaves, fruits, and flowers of *C. monogyna*. Among the phenolic compounds they quantified using HPLC, focusing on the results of chlorogenic acid, which was relevant to current study, the highest amount of chlorogenic acid was found in the leaves (≈10 mg/g), followed by the fruit extract (≈3 mg/g) and flower extract (≈2 mg/g). Sagaradze et al. [34] also performed HPLC analysis on the hydroalcoholic extracts of *C. monogyna* and *C. pentagyna* flowers. Among the compounds they quantified, according to the results of vitexin, rutin, and hyperoside, which were also relevant to this study, the highest amount of hyperoside was found in the extract belonging to *C. monogyna* (≈13 mg/g) flowers, whereas the amounts of vitexin and rutin were higher in *C. pentagyna* than in *C. monogyna*. In parallel with the previous study, in this study, hyperoside in the hydroalcoholic extract of flower-bearing branches belonging to *C. monogyna* (≈17 mg/g) was found to be higher than *C. pentagyna* (≈3 mg/g). Vitexin and rutin were not detected in the flower-bearing branches of *C. monogyna*. Although there is quite enough HPLC study on *Crataegus* species, especially, *C. monogyna*, *C. pentagyna*, and *C. orientalis*, there is no study on different plant parts of *C. turcicus*.

The results of antioxidant activities (DPPH, ABTS, FRAP, and CUPRAC) were expressed as mg trolox equivalent (TE) in 1 g hydroalcoholic extract. According to the results of the DPPH assay, the hydroalcoholic extract of *C. turcicus* flower-bearing branches exhibited the highest antioxidant activity (≈467 mg TE/g) among all the extracts, as observed in the HPLC analysis. The reason for this may have been due to the high amounts of neochlorogenic acid and hyperoside in *C. turcicus*. Froehlicher et al. [26] conducted a study on the DPPH radical scavenging activities of the 80% hydroalcoholic extracts of *C. monogyna* fruits and flowers. The results indicated that the flower extract (≈87 mg TE/g) exhibited greater antioxidant activities compared to the fruit extract (≈16 mg TE/g); these results were found to be similar to the present study. Rocchetti et al. [35] investigated the DPPH radical scavenging activity of a *C. orientalis* leaves extract, which was obtained through different extraction methods, like methanolic maceration, decoction, and infusion. According to their results, the methanolic extract exhibited the highest antioxidant activity, with ≈97 mg TE/g. In this study, the hydroalcoholic extract of *C. orientalis,* which had ≈314 mg total antioxidant content per g showed three times more potent antioxidant effects compared to the methanolic extract in the previous study. Martín-Garcia et al. [36] investigated the DPPH radical scavenging activity of 15 different extracts prepared by varying the acetone–water ratio of *C. monogyna* leaves extract. The values they found ranged from ≈38 to 101 mg TE/g extract. In the current study, the DPPH radical scavenging activity of the *C. monogyna* leaves extract (≈244 mg TE/g) exceeded these values. 

The reason why the hydroalcoholic extract of flowering branches of *C. monogyna* showed the highest antioxidant activity in the results of ABTS radical scavenging activity assay may have been due to the high contents of chlorogenic acid and epicatechin compounds. The extracts of *C. turcicus* leaves (≈365 mg TE/g) was found to have significantly higher antioxidant activity compared to the extracts of *C. orientalis* (≈334 mg TE/g) and *C. monogyna* (≈289 mg TE/g) leaves. Furthermore, the fruit extract of *C. turcicus* (≈131 mg TE/g) was found to have a higher antioxidant activity than the fruit extract of *C. orientalis* (≈119 mg TE/g). Froehlicher et al. [26] conducted a study on the ABTS radical scavenging activities of the fruit and flower extracts of *C. monogyna*. According to the results they obtained, the flower extract (≈26 mg TE/g) showed the highest antioxidant activities compared to the fruit extract (≈13 mg TE/g). Similarly, in the current study, the hydroalcoholic extract of flower-bearing branches belonging to *C. monogyna* (≈457 mg TE/g) exhibited a higher antioxidant activity potential than the fruit extract (≈216 mg TE/g) based on the results of the ABTS radical scavenging activity assay. Rocchetti et al. [33] reported that the antioxidant activity of the methanolic extract obtained from the leaves of the *C. orientalis* was ≈131 mg TE/g. Martín-Garcia et al. [36] conducted a study in which they examined ABTS radical scavenging activity of 15 extracts derived from *C. monogyna* leaves by varying the acetone–water ratio. The observed values ranged from ≈38 mg TE/g to 102 mg TE/g. However, in the present study, the ABTS radical scavenging activity of *C. monogyna* leaves extract surpassed these values (≈289 mg TE/g). Özyürek et al. [37] performed an ABTS radical scavenging activity study using the methanolic extracts of flowers and leaves from *C. monogyna*, *C. orientalis*, and *C. pentagyna*. According to their findings, among the flower extracts, *C. monogyna* had higher antioxidant capacity than other species with ≈98 mg TE/g; also, leaves extract belonging to *C. pentagyna* showed abundant yield compared to other extracts examined.

Moreover, FRAP metal-reducing activities of the extracts belong to leaves, *C. turcicus* (≈227 mg TE/g) showed the highest antioxidant activity, and similarly, among the hydroalcoholic extracts of flower-bearing branches, *C. turcicus* (≈304 mg TE/g) again displayed the highest antioxidant activity. It was determined that the fruit extract of *C. turcicus* (≈106 mg TE/g) had a statistically higher antioxidant effect than the fruit extract of *C. orientalis* (≈67 mg TE/g). Martín-Garcia et al. [36] conducted research, in which they investigated antioxidant activities of 15 extracts obtained from *C. monogyna* leaves, altering the acetone–water ratio. The measured values were found to range from ≈50 to 135 mg TE/g. However, in the current study, *C. monogyna* leaves extract exceeded these values (≈163 mg TE/g extract). Bardakçı et al. [23] investigated the FRAP metal-reducing activities of the fruit extracts from *C. turcicus*, *C. monogyna,* and *C. orientalis*. In their findings, *C. monogyna* (2.83 Mm FeSO_4_ equivalent/g extract) exhibited the highest antioxidant activities, followed by *C. turcicus* (0.86 Mm FeSO_4_ equivalent/g extract) and *C. orientalis* (0.73 Mm FeSO_4_ equivalent/g extract), respectively. In this study, similar to the previous research, when examining *C. turcicus*, *C. monogyna*, and *C. orientalis* fruit extracts, the highest antioxidant effect was observed in the *C. monogyna* (≈129 mg TE/g), followed by *C. turcicus* (≈106 mg TE/g), and then *C. orientalis* (≈67 mg TE/g). Özyürek et al. [37] carried out FRAP assay, using the methanolic extracts obtained from flowers and leaves from *C. monogyna*, *C. orientalis,* and *C. pentagyna*. Based on their findings, the values were determined to range from ≈4 to 37 mg TE/g. 

Bardakçı et al. [23] performed the CUPRAC assay on the fruit extracts of *C. turcicus*, *C. monogyna,* and *C. orientalis*. In their findings, *C. monogyna* (≈560 mg ascorbic acid equivalent/g) demonstrated the highest antioxidant activities, followed by *C. turcicus* (≈255 mg ascorbic acid equivalent/g) and *C. orientalis* (≈190 mg ascorbic acid equivalent/g), respectively. According to the CUPRAC assay results of the current study, consistent with the previous research, *C. monogyna* exhibited the highest antioxidant activities (≈359 mg TE/g), followed by *C. turcicus* (≈277 mg TE/g) and *C. orientalis* (≈222 mg TE/g), respectively. Özyürek et al. [37] conducted a study on the CUPRAC assay by utilizing methanolic extracts derived from flowers and leaves of various *C. monogyna*, *C. orientalis*, and *C. pentagyna* samples, which were collected from different locations. Their results varied according to the place where the plant material was collected, and findings ranged from ≈10 to 95 mg TE/g.

In this study, the anti-inflammatory effect of *Crataegus* species was determined by examining the levels of NO. According to the results obtained, all the samples exhibited anti-inflammatory effects in a concentration-dependent manner. Among all the plant parts studied, the hydroalcoholic extract of *C. pentagyna* leaves showed significantly higher inhibition of NO in LPS-stimulated RAW 264.7 cells compared to the other extracts. This effect may be attributed to the high content of orientin, vitexin, isoquercitrin, and rutin, as detected by the HPLC analysis, in the *C. pentagyna* leaves. Overall, when examining flower-bearing branches, leaves, and fruits of *Crataegus* species, it was determined that the hydroalcoholic extracts of flower-bearing branches and leaves had higher anti-inflammatory effects compared to the fruit extracts. The hydroalcoholic extract of *C. turcicus* leaves was found to have a higher anti-inflammatory effect compared to *C. pentagyna* leaves. Additionally, it was found that the hydroalcoholic extract of flower-bearing branches belonging to *C. turcicus* exhibited higher anti-inflammatory effects compared to the hydroalcoholic extract of flower-bearing branches of *C. monogyna* and *C. pentagyna*. Furthermore, the fruit hydroalcoholic extract of *C. turcicus* showed higher anti-inflammatory effect compared to the fruit extracts of *C. pentagyna* and *C. orientalis*. In a study, the anti-inflammatory effect of the *C. orientalis* was examined. According to the results of the study, it was observed that ethanolic extract of *C. orientalis* had a concentration-dependent anti-inflammatory activity on carrageenan-induced paw edema [38]. In another study conducted by Šavikin et al. [39], the anti-inflammatory activities of hydroalcoholic extracts of *C. orientalis* leaves and fruit were examined. According to the results, both extracts exhibited anti-inflammatory activities. Furthermore, it was found that the hydroalcoholic extracts of the leaves extract had higher anti-inflammatory activities compared to the fruit extract. To calculate the analgesic activity, the inhibition level of PGE_2_ released by LPS-stimulated RAW 264.7 cells was determined for the extracts. According to the results, fruit extracts were found to cause greater decrease in PGE_2_ levels compared to extracts of leaves and flower-bearing branches. Among all samples, the fruit extract *C. turcicus* containing valuable amounts of neochlorogenic acid, procyanidin B2, and epicatechin showed the highest reduction in PGE_2_ levels. Additionally, the hydroalcoholic extract *C. turcicus* leaves exhibited a higher inhibition of PGE_2_ compared to the extracts of *C. monogyna* and *C. pentagyna* leaves. According to the results of the IL-6-releasing inhibition assay, which examined the anti-inflammatory activities, the fruit extract of *C. turcicus* showed a higher inhibition of IL-6-releasing compared to the extracts of *C. orientalis* and *C. pentagyna* fruits. Additionally, it was found that *C. turcicus* exhibited higher IL-6-releasing inhibition among the hydroalcoholic extracts of leaves as well. Lastly, the decrease in TNF-α levels were analyzed, and it was observed that all extracts decreased TNF-α levels. Additionally, the hydroalcoholic extract of *C. turcicus* leaves showed significant anti-inflammatory activities by inhibiting TNF-α release by 93%.

## 4. Materials and Methods

### 4.1. Materials

#### 4.1.1. Plant Material

Among *the Crataegus* species mentioned in Table 5, *C. monogyna* was identified by Assoc. Prof. Dr. Gizem Bulut, and a voucher specimen has been kept at the Herbarium of the Department of Pharmacognosy, Faculty of Pharmacy, Yeditepe University, Istanbul, Türkiye. Other *Crataegus* species (*C. turcicus,* which is an endemic plant to Artvin (Türkiye), *C. pentagyna*, and *C. orientalis*) utilized in this study were collected and authenticated by Prof. Dr. Yüksel Kan. The herbarium specimens have been deposited at Karadeniz Technical University, Forestry Faculty Herbarium (KATO), and Artvin Çoruh University, Forestry Faculty Herbarium (ARTH).

The flower-bearing branches and leaves were dried at room temperature for two weeks under shadow and stored at the same conditions until analysis. Fresh fruits were kept at −20 °C in the freezer and before analysis they were freeze-dried.

#### 4.1.2. Chemicals

All chemicals used in this study were analytical or HPLC grade. Vitexin, vitexin-2″-*O*-rhamnoside, and neochlorogenic acid were purchased from Aktin Chemicals (Chengdu, China); epicatechin was bought from Biosynth (Staad, Switzerland); ammonium acetate and copper sulfate were provided from Carlo Erba Reagents (Val de Reuil, France); vanillin, 2,4,6-Tri(2-pyridyl)-s-triazine (TPTZ), sulfanilamide, sodium acetate trihydrate, acetic acid, ammonium molybdate, sulfuric acid (98%), dimethyl sulfoxide, and sodium nitrite were acquired from Honeywell Research Chemicals (Morris Plains, NJ, USA); hyperoside and quercetin were obtained from HWI (Rülzheim, Germany); n-propanol was bought from Interlab (İstanbul, Türkiye); HPLC grade acetonitrile, iron (III) chloride (FeCl_3_), and iron (II) sulfate heptahydrate were purchased from J.T. Baker (Phillipsburg, NJ, USA); cyanidin-3-*O*-glucoside and cyanidin chloride were acquired from Phytolab (Vestenbergsgreuth, Germany); sodium acetate, ethyl methyl ketone, aluminum chloride, *o*-phosphoric acid, polyethylene glycol 400 (PEG 400), potassium chloride, rutin, potassium persulphate, 2,2-diphenyl-1-picrylhydrazyl (DPPH), isoquercitrin, 2,2′-azino-bis(3-ethylbenzothiazoline-6-sulfonic acid diammonium salt (ABTS), sodium carbonate (Na_2_CO_3_), ferric chloride hexahydrate, formic acid, procyanidin B2, ethanol absolute, gallic acid, trolox, caffeic acid, (+)-catechin hydrate, Folin–Ciocalteu Reagent, neocuproine, ethyl acetate, hydrochloric acid (36.5%), methanol absolute, chlorogenic acid, glacial acetic acid, protocatechuic acid, and magnesium chloride hexahydrate were supplied from Merck (Darmstadt, Germany); and orientin was provided from TRC Corp. (Burlington, ON, Canada).

The RAW 264.7 mouse macrophages (ATCC^®^ TIB-71 TM) utilized in this research were obtained from ATCC (ATCC, Manassas, VA, USA). These cells were cultivated in complete medium, which consisted of Dulbecco’s modified Eagle’s medium (DMEM) (Gibco, Thermo Fisher Scientific, Waltham, MA, USA), supplemented with 10% fetal bovine serum (Sigma Aldrich, St. Louis, MI, USA), 10% FBS, 1% penicillin (10,000 units/mL), and streptomycin (10.000 mg/mL) (Gibco, Thermo Fisher Scientific, Waltham, MA, USA). They were maintained at a temperature of 37 °C with 5% carbon dioxide in a humidified atmosphere [40]. The cells were sub-cultured once they reached a confluence of 80–90%.

### 4.2. Methods

#### 4.2.1. Sample Extraction and Preparation of Sample Solution

Dried flower-bearing branches, leaves, and freeze-dried fruits of *Crataegus* species were ground with a laboratory mill (Arcelik, K3104, İstanbul, Türkiye), then 5 g of powdered plant parts were extracted with 100 mL of 70% ethanol_(aq)_ using an ultrasonic bath (Bandelin 156BH, Germany) for 30 min [41]. The solutions obtained were filtered with 0.2 mm thick filter paper (Macherey-Nagel, Düren, Germany), and then ethanol was evaporated by using a rotary evaporator (Heidolph, Schwabach, Germany) at 45 °C and 80 mbar pressure conditions. The remaining aqueous part was frozen at −20 °C overnight and then lyophilized at −55 °C and 0.2 mbar pressure. After the lyophilization process, obtained hydroalcoholic extracts were transferred to amber bottles and stored in a refrigerator at +4 °C throughout the experiment.

Each lyophilized extract (100 mg) was accurately weighed and dissolved with 5 mL of 70% ethanol_(aq)_ in an ultrasonic bath for 15 min, and suspended particles were removed by filtration through a 0.45 µm RC-membrane filter (Minisart, Sartorius Stedim Biotech, Goettingen, Germany). The final concentration of each sample stock test solution was 20 mg/mL. The stock solution was further diluted according to the desired concentration to be used in subsequent experiments as a sample test solution.

#### 4.2.2. Preparation of Standard Solutions

All standard solutions were prepared in methanol. Stock solutions of standards (protocatechuic acid, vitexin, isoquercitrin, orientin, hyperoside, neochlorogenic acid, chlorogenic acid, vitexin-2″-*O*-rhamnoside, rutin, epicatechin, procyanidin B2, and cyanidin-3-*O*-glucoside) were prepared at a concentration of 200 μg/mL. These stock solutions were further diluted for HPTLC and HPLC analyses.

Standards used to prepare the calibration curve to estimate the contents of total phenolic, flavonoid, anthocyanin, and proanthocyanidin were gallic acid, quercetin, cyanidin chloride, and cyanidin-3-*O*-glucoside, respectively were prepared at the concentration of 200 μg/mL. In addition, trolox standard solution which was used to evaluate the antioxidant activity was prepared at the concentration of 200 μg/mL. These stock solutions were serial diluted to obtain calibration curves.

#### 4.2.3. Total Phenolic Content (TPC) Assay

In each well of a 96-well plate, 100 μL of 7.5% Na_2_CO_3_, 25 μL of sample solution (400 μg/mL used for hydroalcoholic extracts of fruits and 100 μg/mL for hydroalcoholic extracts of flower-bearing branches and leaves), different concentrations of gallic acid standard solutions (4–125 μg/mL) and blank (methanol) were added. Then, 100 μL of 10% Folin–Ciocalteu reagent was added to these mixtures and incubated in the dark for 30 min at room temperature. After that, absorbance was measured at 760 nm by using a microplate reader (Multiskan Go, Thermo Scientific, Waltham, MA, USA) [42]. The results were expressed as mg gallic acid equivalent (GAE) per g of hydroalcoholic extract (mg GAE/g).

#### 4.2.4. Total Flavonoid Content (TFC) Assay

In each well (96-well microplate), which contained 30 μL of sample test solution (concentration of 2 mg/mL used for hydroalcoholic extracts of fruits and 4 mg/mL used for the other extracts) or methanol as blank or quercetin standard solutions (4–125 μg/mL), 30 μL of 10% aluminum chloride, 30 μL of 1 M sodium acetate, and 150 μL water were added sequentially. After the incubation period (15 min), absorbance was measured at 415 nm using a microplate reader [43]. The total flavonoid content of the extracts was indicated as mg quercetin equivalent (QE) per g hydroalcoholic extract (mg QE/g).

#### 4.2.5. Total Proanthocyanidin Content (TPAC) Assay

TPAC assay was performed as follows: 125 μL of 1% vanillin (in methanol) and 125 μL of 25% sulfuric acid (in methanol) were added into each well of 96-well microplate containing 50 μL of sample test solution (4 mg/mL), blank (methanol), or cyanidin chloride standard solutions (4–125 μg/mL). Then, the mixture was incubated for 15 min at 30 °C in the dark. Absorbance was read at 500 nm as soon as the incubation period was over [44]. The results were mentioned as mg cyanidin chloride equivalent per g hydroalcoholic extract (mg CCE/g).

#### 4.2.6. Total Anthocyanin Content (TAC) Assay

To perform the TAC assay, 160 μL of pH_1_ (prepared with 25 mM potassium chloride and pH adjusted with diluted hydrochloric acid or sodium hydroxide) and pH_4.5_ buffer solution (prepared using 400 mM sodium acetate and pH modified with diluted hydrochloric acid or sodium hydroxide) were added into 40 μL of hydroalcoholic extracts of fruits (4 mg/mL) and different concentrations of cyanidin-3-*O*-glucoside standard solutions (6–200 μg/mL) separately. The intensity of red color formation was measured at 520 nm and 700 nm [45]. The absorbances obtained in the total anthocyanin experiment were calculated using the formula: Absorbance (A): (A_520_−A_700_)_pH1_−(A_520_−A_700_)_pH4.5._

The results were given as mg cyanidin-3-*O*-glucoside equivalent per g hydroalcoholic extract (mg C3GE/g).

#### 4.2.7. HPTLC Method

Major flavonoids, phenolic acids, and anthocyanins in the *Crataegus* species were qualitatively screened by HPTLC method. Standard mixtures and 5 µL (also applied 10 µL to detect anthocyanins) of 20 mg/mL test solutions containing each of hydroalcoholic extracts belonging to *Crataegus* species were applied to glass-backed HPTLC plates (Merck, Darmstadt, Germany) coated with silica gel 60 F_254_ as 10 mm bands by a semi-automatic sample dispenser Linomat V (Camag, Muttenz, Switzerland). 

As indicated in Table 6, different developing solvent systems were utilized for the separation of subclasses of phenolic compounds. For separation of phenolic acids (protocatechuic acid, neochlorogenic acid, and chlorogenic acid), flavones (vitexin, orientin, and vitexin-2″-*O*-rhamnoside), flavonols (isoquercitrin and hyperoside), and anthocyanins (cyanidin-3-*O*-glucoside), a solvent system containing ethyl acetate–methyl ethyl ketone-formic acid–water (5:3:1:1, *v*/*v*/*v*/*v*) was used whereas for flavanols (catechin and procyanidin B2), an upper phase of ethyl acetate-formic acid–water (10:1:4, *v*/*v*/*v*) solvent mixture was applied. Plates were developed up to 7 cm in the saturated (20 min) Automatic Developing Chamber 2 (ADC2, Camag). The relative humidity was adjusted by using a saturated magnesium chloride hexahydrate solution.

After the development process, plates were automatically dried for 5 min by ADC2. HPTLC plate encoded as Plate 1 was heated at 105 °C for 3 min via plate heater, then derivatized by using an immersion device (Camag) with natural product (0.5% 2-aminoethyl diphenylborinate prepared in ethyl acetate) and PEG 400 (5% prepared in dichloromethane) solutions, respectively. TLC visualizer (Camag) was used to capture the images of the derivatized plates under UV (366 nm). HPTLC plate coded as Plate 2 was derivatized through vanillin/sulfuric acid reagent (mixing 5 g vanillin with 475 mL ethanol and 25 mL sulfuric acid). Derivatization was not performed for HPTLC Plate 3, as anthocyanins were colored on the plate without using a derivatization reagent. HPTLC images of Plate 2 and 3 were documented after capturing the plates at white light. The winCATS program operated all the instruments (Camag, Version 128 1.4.8.2031).

#### 4.2.8. HPLC Method

The Agilent 1260 Infinity HPLC system (Darmstadt, Germany), which comprises an Agilent ChemStation software (version C.01.07 SR3), quaternary pump (G1311B), auto-sampler (G1329B), thermostatted column compartment (G1316A), and diode array detector (G4212B), was used during the analysis. The HPLC method was applied according to previously published method by Ying et al. [46] with slight modifications. The analysis was performed at 25 °C on a reverse-phase analytical column (Agilent Zorbax C-18 Column (4.6 mm × 250 mm, 5-μm particle size)). Mobile phase A was *o*-phosphoric acid–water (0.5:99.5, *v*/*v*) and B was acetonitrile–tetrahydrofuran (95:5, *v*/*v*). The mobile phases were filtered and degassed before analysis. The gradient elution in HPLC was applied as 5–10% B (0–20 min), 10–13.5% B (20–45 min), 13.5–75% B (45–47 min), 75% B (47–48 min), and 75–5% B (48–50 min). The flow rate of the mobile phase was 1 mL/min. The injection volume of the analytes was 10 μL.

For quantitative analysis, protocatechuic acid, hyperoside, rutin, and isoquercitrin were detected at 260 nm, whereas procyanidin B2 and epicatechin were identified at 280 nm. In addition, 330 nm was used to monitor neochlorogenic acid and chlorogenic acid. Vitexin and vitexin-2″-*O*-rhamnoside were evaluated at 340 nm, whereas orientin was monitored at 350 nm.

The linearity of the calibration curves was obtained by using seven different working standard solutions in between 5 to 50 µg/mL prepared from the stock solutions of protocatechuic acid, neochlorogenic acid, chlorogenic acid, procyanidin B2, epicatechin, orientin, vitexin, hyperoside, and isoquercitrin and 10 to 100 µg/mL for vitexin-2″-*O*-rhamnoside and rutin. The regression equations and coefficient of determination (*r*^2^) are stated in Table 7. For the calculation of the limit of quantification (LOQ) and limit of detection (LOD) values, calibration curves were obtained on consecutive days and the obtained values were calculated by using the following equations: 10 × (SD/S) and 3 × (SD/S), respectively (Table 7). 

In addition, the known concentrations of the standards were compared with the measured results obtained by using the calibration curve. The closeness of the obtained results by the HPLC method to their theoretical values were expressed as percentages, presented in Table 8. 

### 4.3. Bioactivity Assays

#### 4.3.1. Antioxidant Activity

##### DPPH Radical-Scavenging Activity Assay

DPPH (2,2-diphenyl-1-picrylhydrazyl) radical scavenging activity was performed with slight changes according to Blois [47]. In this experiment, 280 μL of methanolic DPPH reagent, which was prepared at 0.1 mM (absorbance ∼0.7) concentration, was added to 20 μL of each sample test solution (between 100–400 μg/mL), blank (methanol), and trolox (3–200 μg/mL) standard solutions. After 30 min of incubation in a dark environment, absorbance was read at 520 nm with a microplate reader. The outcomes of all antioxidant experiments (DPPH, ABTS, CUPRAC, and FRAP) were represented as mg trolox equivalents (TE) in 1 g dry extract (mg TE/g).

##### ABTS Radical-Scavenging Activity Assay

ABTS (2,2′-azino-bis-(3-ethylbenzothiazoline-6-sulfonic) acid) radical-scavenging activity was carried out in a 96-well plate by using the procedure by Re et al. with slight modifications [48]. A 20 μL sample (100 μg/mL), blank (methanol), or trolox (4–125 μg/mL) standard solution was mixed with 280 μL ABTS reagent (7 × 10^−3^ M ABTS and 2.45 × 10^−3^ M potassium sulfate were mixed in equal volumes. The mixture was kept at room temperature in the dark for 12–16 h, then diluted with methanol at the ratio of 1:10, *v*/*v*) with an absorbance of ∼0.7. Immediately after the incubation period of 6 min, absorbances of color changes were measured at a wavelength of 734 nm wavelength. 

##### CUPRAC Assay

A cupric-reducing antioxidant capacity assay of the samples was performed in a 96-well plate according to the method of Apak et al. with some modifications [49]. Initially, 85 μL volumes of 10^−2^ M copper (II) sulfate pentahydrate, 7.5 × 10^−3^ M neocuproine, and ammonium acetate buffer (pH 7) were added into the wells, respectively, and a blue structure was formed. After, 43 μL of sample (1 mg/mL), blank (water), or standard solution (4–200 μg/mL) and 51 μL of distilled water were added to the blue color mixture. The absorbance of the reaction, which changed from blue to yellow after an incubation period of 20 min at 50 °C, was read at a wavelength of 450 nm. 

##### FRAP Assay

A ferric-reducing antioxidant power assay was performed spectrophotometrically, according to Benzie and Strain, with minor modifications [50]. In total, 280 μL of FRAP reagent (2 × 10^−2^ M FeCl_3_, 1 × 10^−2^ M TPTZ and sodium acetate buffer (pH 3.6) solutions were mixed with the ratio of 1:1:10, respectively), which was prepared freshly just before the analysis, was added to 20 μL of sample (100–400 μg/mL), blank (water), or trolox (4–125 μg/mL) standard solutions. The absorbance of the formed blue structure after 6 min of incubation was evaluated at a wavelength of 595 nm.

#### 4.3.2. Anti-Inflammatory Activity

##### Cell Viability Assay

To determine the effect of *Crataegus* species on cell viability, an MTT (3-(4,5-dimethylthiazol-2-yl)-2,5-diphenyltetrazolium bromide) assay was used. The RAW 264.7 cells were initially seeded in 96-well plates at a density of 5 × 10^4^ cells per well for 24 h in complete medium. The cells were then treated with various concentrations of the *Crataegus* extracts (ranging from 0.125 to 1 mg/mL) for 24 h. Following this, the MTT reagent (0.5 mg/mL in PBS) (Sigma Aldrich, St. Louis, MI, USA) was added to the cells and incubated for 2 h at 37 °C with 5% CO_2_. After removing the culture supernatants, 100 µL of isopropanol was added to each well, and the absorbance at 570 nm was measured using a microplate reader (Thermo Fisher ScientificTM, Inc., Waltham, MA, USA). The cell viability assay was conducted three times, and each assay was performed in triplicate (*n* = 9 in three separate experiments). The cell viability of cultures treated with gels of less than 70% compared to untreated control cultures (medium group) was considered cytotoxic [51]. The percentage of cell viability was calculated by using the following equation:

Viability % = (Absorbance_Treatment group_)/(Absorbance_Control_) × 100%

##### Evaluation of Anti-Inflammatory Activity

The inhibition of NO production was assessed by measuring NO levels in the cell culture medium using the Griess reagent (0.1% n-(1-naphthyl)-ethylenediamine dihydrochloride in 5% phosphoric acid and 1% sulfanilamide) [52]. RAW 264.7 cells were plated in a 96-well plate at a density of 5 × 10^4^ cells/well and incubated for 24 h at 37 °C with 5% CO_2_. After pre-treating the cells with various concentrations of the hydroalcoholic extracts of *Crataegus* species (0.125–0.25–0.5–1 mg/mL) for 2 h and stimulating them with 1 µg/mL of LPS (Lipopolysaccharide from *Escherichia coli* 0111: B4, Sigma, USA) for an additional 22 h, the cell culture supernatant was collected. The cell culture supernatants were collected after 24 h for NO analysis. Nitrite concentrations were measured using Griess’ reagent. The supernatant was mixed with an equal volume of Griess reagent in a 96-well plate for 10 min at room temperature in the dark. The color development corresponding to NO level was assessed at 540 nm using a microplate reader (Thermo Fisher ScientificTM, Inc., Waltham, MA, USA). The nitrite concentrations were determined using a sodium nitrite (Fluka Chemika-BioChemika, Ronkonkoma, N.Y., Buchs, Switzerland) standard curve. The researchers used indomethacin (100 mM) (Sigma Aldrich, St. Louis, MI, USA) as a positive control [53]. 

##### Enzyme-Linked Immunosorbent Assay (ELISA)

After overnight culture in a 96-well plate (5 × 10^4^ cells/well), the cells were pre-treated with *Crataegus* species for 2 h and 1 µg/mL lipopolysaccharide for an additional 22 h, and the culture supernatant from each well was collected at the end of scheduled experiments and used to measure TNF-α (Invitrogen, Carlsbad, CA, USA), IL-6 (Invitrogen, USA) [54] and PGE_2_ (Abcam PGE_2_ ELISA Kit, Cambridge, UK) [53] concentration by ELISA, according to the manufacturer’s instructions.

### 4.4. Statistical Analysis

Each test was performed thrice, the average value and standard deviation (SD) were calculated, and the results were expressed as average ± SD. Microsoft Excel 2013 and Minitab 17 were used during the analyses. After performing one-way analysis of variance (one-way ANOVA), the statistical significance was considered as *p* ≤ 0.05. Statistical analysis of cell culture studies was performed by GraphPadPrism 8.0 software (LaJolla, CA, USA) with one-way ANOVA. 

## 5. Conclusions

Overall, flower-bearing branches, leaves, and fruits of *C. turcicus,* which is a plant endemic to Türkiye, were compared with other well-known *Crataegus* species (*C. monogyna*, *C. pentagyna*, and *C. orientalis*) in terms of chemical composition and bioactivity studies. It can be concluded that *C. turcicus* may have potential to be an alternative to the officinal *Crataegus* species in the market as a food supplement. The cultivation of *C. turcicus* should be considered for reducing the over-exploitation of wild populations.

## Figures and Tables

**Table 1:** Total phenolic, flavonoid, proanthocyanidin, and anthocyanin contents of hydroalcoholic extracts belong to different parts of *Crataegus* species.

Samples	Parts	TPC ^1^	TFC ^2^	TPAC ^3^	TAC ^4^
*C. turcicus*	flower-bearing branches	239.09 ± 9.77 ^DE^	22.15 ± 1.37 ^CD^	30.73 ± 0.54 ^A^	-
	leaves	227.57 ± 12.05 ^DE^	34.26 ± 2.77 ^B^	20.98 ± 0.05 ^C^	-
	fruits	69.43 ± 5.9 ^G^	1.54 ± 0.17 ^F^	12.96 ± 0.62 ^F^	6.47 ± 0.45 ^B^
*C. monogyna*	flower-bearing branches	301.77 ± 21.8 ^CD^	20.49 ± 0.69 ^D^	30.11 ± 1.14 ^A^	-
	leaves	169.66 ± 12.34 ^EF^	20.54 ± 1.33 ^D^	17.22 ± 1.31 ^DE^	-
	fruits	116.24 ± 10.83 ^FG^	6.08 ± 0.53 ^EF^	15.16 ± 0.43 ^EF^	2.02 ± 2.02 ^C^
*C. orientalis*	flower-bearing branches	287.92 ± 37.02 ^CD^	20.72 ± 0.53 ^D^	28.69 ± 3.26 ^A^	-
	leaves	874.06 ± 74.94 ^A^	37.47 ± 3.48 ^B^	20.01 ± 0.47 ^CD^	-
	fruits	42.12 ± 4.71 ^G^	8.37 ± 0.98 ^E^	7.46 ± 0.50 ^G^	0.24 ± 0.24 ^D^
*C. pentagyna*	flower-bearing branches	368.94 ± 7.78 ^BC^	26.92 ± 1.18 ^C^	24.67 ± 1.07 ^B^	-
	leaves	394.66 ± 35.05 ^B^	63.67 ± 4.71 ^A^	20.17 ± 1.20 ^CD^	-
	fruits	91.00 ± 2.77 ^FG^	9.87 ± 0.67 ^E^	18.76 ± 0.51 ^CD^	15.45 ± 15.45 ^A^

TPC: Total Phenolic Content; TFC: Total Flavonoid Content; TPAC: Total Proanthocyanidin Content; TAC: Total Anthocyanin Content. ^1^ mg gallic acid equivalent per g hydroalcoholic extract (mg GAE/g). ^2^ mg quercetin equivalent per g hydroalcoholic extract (mg QE/g). ^3^ mg cyanidin chloride equivalent per g hydroalcoholic extract (mg CCE/g). ^4^ mg cyanidin-3-*O*-glucoside equivalent per g hydroalcoholic extract (mg C3GE/g). Different letters in the same column indicate significantly different values at *p* ≤ 0.05.

**Table 2:** Quantitative results of the investigated compounds in *Crataegus* species by HPLC.

	Flower-Bearing Branches				Leaves				Fruits			
	C.T.	C.M.	C.O.	C.P.	C.T.	C.M.	C.O.	C.P.	C.T.	C.M.	C.O.	C.P.
Protocatechuic acid	0.15 ± 0.01 ^H^	0.18 ± 0.01 ^H^	0.39 ± 0.01 ^F^	0.43 ± 0.03 ^E^	0.35 ± 0.01 ^G^	0.77 ± 0.00 ^B^	0.55 ± 0.01 ^C^	0.88 ± 0.00 ^A^	0.04 ± 0.01 ^I^	n.d.	n.d.	0.49 ± 0.01 ^D^
Neochlorogenic acid	18.18 ± 0.56 ^C^	21.90 ± 0.06 ^B^	7.82 ± 0.22 ^DE^	50.32 ± 4.02 ^A^	n.d.	4.64 ± 0.04 ^EF^	3.26 ± 0.07 ^FG^	4.64 ± 0.03 ^EF^	6.90 ± 0.02 ^DE^	1.26 ± 0.01 ^FG^	0.86 ± 0.04 ^G^	9.17 ± 0.16 ^D^
Chlorogenic acid	21.61 ± 0.61 ^E^	45.52 ± 0.50 ^A^	12.12 ± 0.13 ^G^	15.69 ± 0.75 ^F^	29.60 ± 0.04 ^D^	40.89 ± 0.44 ^B^	13.73 ± 0.39 ^G^	32.35 ± 0.02 ^C^	0.93 ± 0.01 ^H^	2.14 ± 0.07 ^H^	0.55 ± 0.00 ^H^	0.71 ± 0.01 ^H^
Procyanidin B2	3.67 ± 0.07 ^F^	7.15 ± 0.05 ^B^	5.48 ± 0.19 ^D^	6.44 ± 0.04 ^C^	1.81 ± 0.09 ^I^	2.03 ± 0.09 ^HI^	2.92 ± 0.17 ^G^	1.97 ± 0.10 ^HI^	4.56 ± 0.05 ^E^	11.65 ± 0.17 ^A^	3.24 ± 0.14 ^G^	2.29 ± 0.16 ^H^
Epicatechin	3.59 ± 0.03 ^FG^	17.56 ± 0.27 ^A^	11.78 ± 0.75 ^C^	13.83 ± 0.11 ^B^	2.83 ± 0.10 ^G^	5.95 ± 0.11 ^E^	3.82 ± 0.51 ^F^	7.30 ± 0.02 ^D^	7.92 ± 0.06 ^D^	13.24 ± 0.18 ^B^	2.89 ± 0.12 ^G^	13.29 ± 0.21 ^B^
Orientin	0.08 ± 0.07 ^D^	n.d.	n.d.	1.54 ± 0.02 ^B^	0.47 ± 0.03 ^C^	n.d.	n.d.	13.49 ± 0.03 ^A^	n.d.	n.d.	n.d.	n.d.
Vitexin	0.90 ± 0.03 ^E^	n.d.	1.09 ± 0.08 ^D^	0.30 ± 0.05 ^F^	3.61 ± 0.14 ^B^	n.d.	1.77 ± 0.01 ^C^	4.98 ± 0.02 ^A^	n.d.	n.d.	n.d.	n.d.
Vitexin-2″-O-rhamnoside	4.92 ± 0.18 ^G^	26.50 ± 0.70 ^C^	13.93 ± 0.10 ^E^	1.70 ± 0.09 ^H^	12.83 ± 0.14 ^F^	76.03 ± 0.36 ^A^	49.41 ± 0.92 ^B^	19.63 ± 0.02 ^D^	n.d.	n.d.	n.d.	n.d.
Hyperoside	7.81 ± 0.22 ^E^	17.29 ± 0.01 ^A^	11.54 ± 0.31 ^C^	2.84 ± 0.03 ^H^	4.41 ± 0.22 ^G^	10.09 ± 0.55 ^D^	16.25 ± 0.29 ^B^	n.d.	0.45 ± 0.00 ^J^	5.61 ± 0.15 ^F^	2.23 ± 0.01 ^HI^	1.68 ± 0.02 ^I^
Rutin	1.83 ± 0.08 ^C^	0.34 ± 0.03 ^G^	1.33 ± 0.11 ^DE^	1.80 ± 0.01 ^CD^	5.27 ± 0.32 ^B^	1.67 ± 0.10 ^CD^	5.82 ± 0.25 ^A^	6.03 ± 0.37 ^A^	0.84 ± 0.00 ^F^	n.d.	n.d.	0.96 ± 0.02 ^EF^
Isoquercitrin	n.d.	3.07 ± 0.11 ^F^	6.30 ± 0.24 ^C^	5.12 ± 0.18 ^D^	n.d.	4.03 ± 0.02 ^E^	11.09 ± 0.23 ^B^	12.94 ± 0.06 ^A^	n.d.	2.18 ± 0.00 ^G^	1.28 ± 0.45 ^H^	2.02 ± 0.02 ^G^

C.T.: *C. turcicus*, C.M.: *C. monogyna*, C.O.: *C. orientalis*, C.P.: *C. pentagyna*. The quantitative results are given as mg/g hydroalcoholic extract. n.d.: not detected. Different letters in the same row indicate significantly different values at *p* ≤ 0.05.

**Table 3:** Total antioxidant capacity of hydroalcoholic extracts belonging to different parts of *Crataegus* species.

Samples	Parts	DPPH	ABTS	FRAP	CUPRAC
		mg TE/g			
*C. turcicus*	flowering branches	466.59 ± 8.97 ^A^	380.78 ± 20.11 ^BCD^	304.48 ± 6.89 ^B^	755.42 ± 16.76 ^CD^
	leaves	346.65 ± 7.80 ^C^	365.47 ± 25.53 ^CD^	226.50 ± 8.66 ^D^	692.83 ± 39.22 ^D^
	fruits	129.93 ± 4.17 ^F^	130.78 ± 4.63 ^G^	105.51 ± 6.30 ^H^	277.03 ± 20.28 ^G^
*C. monogyna*	flowering branches	319.29 ± 12.60 ^CD^	457.27 ± 38.03 ^A^	371.52 ± 9.04 ^A^	852.09 ± 21.16 ^A^
	leaves	243.63 ± 8.18 ^E^	289.05 ± 10.26 ^E^	162.66 ± 3.33 ^F^	568.10 ± 10.41 ^E^
	fruits	132.89 ± 20.32 ^F^	215.97 ± 24.11 ^F^	128.81 ± 11.67 ^G^	359.33 ± 11.57 ^F^
*C. orientalis*	flowering branches	383.98 ± 19.93 ^B^	400.34 ± 14.59 ^ABC^	271.72 ± 5.84 ^C^	779.56 ± 41.05 ^BC^
	leaves	313.59 ± 13.11 ^CD^	334.43 ± 15.68 ^DE^	198.72 ± 7.68 ^E^	596.73 ± 32.51 ^E^
	fruits	70.04 ± 2.70 ^G^	119.45 ± 11.95 ^G^	66.52 ± 3.28 ^I^	222.13 ± 11.82 ^GH^
*C. pentagyna*	flowering branches	411.07 ± 3.34 ^B^	442.72 ± 32.10 ^AB^	272.86 ± 9.44 ^C^	847.94 ± 30.56 ^AB^
	leaves	296.23 ± 10.91 ^D^	397.07 ± 29.02 ^ABCD^	217.48 ± 11.91 ^DE^	553.50 ± 28.91 ^E^
	fruits	160.02 ± 14.22 ^F^	148.89 ± 5.34 ^G^	163.89 ± 2.24 ^F^	180.87 ± 0.61 ^H^

Different letters in the same column indicate significantly different values at *p* ≤ 0.05.

**Table 4:** Effects of hydroalcoholic extracts of *Crataegus* species on the viability of RAW 264.7 macrophage cells and the effects of these extracts on nitrite levels and % nitrite inhibition in RAW 264.7 cells stimulated with 1 μg/mL LPS.

Samples	Groups	Concentration (mg/mL)	Cell Viability (%)	Nitrite Level (µM)	Nitrite Inhibition (%)
	Ctrl		117.13 ± 1.75	2.15 ± 2.27	-
	Ctrl + LPS		101.12 ± 2.14	64.17 ± 0.84	-
	Indomethacin	100 µM	92.92 ± 1.47	32.20 ± 0.95 *	49.83 ± 1.39
*C. turcicus*	flower-bearing branches	0.125	115.61 ± 0.73	63.49 ± 1.18	1.06 ± 0.88
		0.25	107.82 ± 1.93	61.33 ± 0.81	4.42 ± 0.58
		0.5	104.06 ± 1.47	55.04 ± 1.03	14.22 ± 2.23
		**1**	102.15 ± 1.47	13.86 ± 1.41 *	78.39 ± 2.31
	leaves	0.125	107.50 ± 0.71	61.15 ± 1.77	4.71 ± 2.90
		0.25	95.32 ± 2.83	54.11 ± 0.49	15.67 ± 1.18
		0.5	83.70 ± 1.07	48.01 ± 1.15 *	25.19 ± 1.62
		**1**	79.97 ± 3.61	13.99 ± 1.30 *	78.19 ± 2.20
	fruits	0.125	117.56 ± 0.94	61.58 ± 1.98	4.05 ± 2.13
		0.25	110.97 ± 1.72	58.62 ± 2.53	8.65 ± 1.05
		0.5	102.83 ± 1.64	43.06 ± 0.88 *	32.90 ± 0.98
		**1**	96.66 ± 4.04	32.75 ± 2.26 *	48.94 ± 3.99
*C. monogyna*	flower-bearing branches	0.125	108.40 ± 1.18	58.99 ± 1.37	8.09 ± 1.12
		0.25	99.89 ± 0.63	55.41 ± 1.70	13.66 ± 2.45
		0.5	93.50 ± 1.21	50.22 ± 3.70	21.77 ± 1.93
		**1**	86.20 ± 0.65	21.27 ± 2.08 *	66.87 ± 2.13
	leaves	0.125	106.40 ± 0.23	59.67 ± 0.98	7.02 ± 0.48
		0.25	96.79 ± 0.98	52.51 ± 1.57	18.16 ± 2.33
		0.5	89.85 ± 1.44	43.00 ± 0.32 *	32.98 ± 1.32
		**1**	84.30 ± 2.10	13.80 ± 0.47 *	78.49 ± 0.92
	fruits	0.125	107.77 ± 1.53	57.69 ± 1.23	10.09 ± 2.50
		0.25	101.11 ± 0.84	47.63 ± 3.80 *	25.75 ± 1.42
		0.5	91.91 ± 1.48	37.51 ± 2.68 *	41.52 ± 1.83
		**1**	85.39 ± 2.06	23.12 ± 1.49 *	63.98 ± 2.05
*C. orientalis*	flower-bearing branches	0.125	114.39 ± 1.55	56.64 ± 1.19	11.71 ± 2.95
		0.25	110.37 ± 1.04	51.52 ± 0.67	19.71 ± 1.21
		0.5	103.35 ± 2.97	44.54 ± 0.60 *	30.58 ± 1.23
		**1**	95.53 ± 0.95	11.09 ± 2.23 *	82.71 ± 2.59
	leaves	0.125	108.88 ± 0.69	63.12 ± 0.60	1.63 ± 1.42
		0.25	102.43 ± 1.15	58.06 ± 2.15	9.51 ± 2.13
		0.5	98.61 ± 1.63	54.67 ± 1.03	14.81 ± 1.32
		**1**	93.55 ± 1.12	10.65 ± 0.57 *	83.40 ± 0.78
	fruits	0.125	110.72 ± 0.89	60.72 ± 2.19	5.40 ± 2.55
		0.25	104.69 ± 1.64	52.57 ± 0.65	18.07 ± 1.86
		0.5	100.34 ± 1.76	51.02 ± 0.47	20.48 ± 1.04
		**1**	90.10 ± 0.26	45.96 ± 1.85 *	28.38 ± 2.62
*C. pentagyna*	flower-bearing branches	0.125	112.26 ± 1.32	58.49 ± 0.75	8.84 ± 1.97
		0.25	109.34 ± 2.51	53.06 ± 1.60	17.33 ± 1.47
		0.5	99.33 ± 0.92	48.62 ± 2.51 *	24.23 ± 2.06
		**1**	83.23 ± 0.52	27.44 ± 1.28 *	57.23 ± 2.05
	leaves	0.125	115.10 ± 1.54	48.68 ± 3.06	24.18 ± 1.78
		0.25	108.45 ± 1.64	43.62 ± 1.19	32.02 ± 2.14
		0.5	99.85 ± 1.98	30.22 ± 1.77 *	52.88 ± 2.28
		**1**	88.03 ± 1.29	8.19 ± 1.79 *	87.24 ± 2.81
	fruits	0.125	106.00 ± 0.84	63.19 ± 0.67	1.54 ± 0.42
		0.25	100.88 ± 1.77	60.41 ± 1.47	5.87 ± 1.67
		0.5	96.09 ± 0.88	54.48 ± 1.34	15.10 ± 2.01
		**1**	85.62 ± 1.37	41.70 ± 0.49 *	35.01 ± 0.99

Ctrl: Control group treated with DMEM; Ctrl + LPS: Control group only stimulated with LPS; LPS: Lipopolysaccharides from *E. coli*; and IND: Indomethacin (100 µM). Statistically significant differences were indicated for each compound vs. LPS (* *p* < 0.0001).

**Table 5:** Detailed information on *Crataegus* species used in the study.

*Crataegus* Species	Parts	Collection Month	Collection Place	Altitude	Herbarium Number
*Crataegus turcicus* Dönmez	Flower-bearing branches	June 2020	Şavşat, Artvin	1650 m	ARTH 13587
	Leaves	May 2020			
	Fruits	October 2020			
*Crataegus monogyna* Jacq.	Flower-bearing branches	May 2020	Ataşehir, Istanbul	195 m	YEF 20048
	Leaves	May 2020			
	Fruits	September 2020			
*Crataegus orientalis* Pall. ex M.Bieb.	Flower-bearing branches	May 2020	Ardanuç, Artvin	1650 m	ARTH 13585
	Leaves	May 2020			
	Fruits	October 2020			
*Crataegus pentagyna* Waldst. & Kit. ex Willd.	Flower-bearing branches	May 2020	Ardanuç, Artvin	1035 m	KATO 19304
	Leaves	May 2020			
	Fruits	October 2020			

**Table 6:** Detailed information on the investigated compounds and parameters used in HPTLC.

Plate No.	Investigated Compounds	Sub-Classes	Applied Concentration	Applied Volume	Mobile Phase	Derivatization Reagents	Visualization
1	Protocatechuic acid	Phenolic acid	50 µg/mL	10 µL	5:3:1:1 (*v*/*v*)	NP/PEG	366 nm
	Vitexin	Flavone C-glycoside					
	Isoquercitrin	Flavonol glycoside					
	Orientin	Flavone C-glycoside					
	Hyperoside	Flavonol glycoside					
	Neochlorogenic acid	Phenolic acid					
	Chlorogenic acid	Phenolic acid					
	Vitexin-2″-*O*-rhamnoside	Flavone C-glycoside					
	Rutin	Flavonol glycoside					
2	Epicatechin	Flavanol	100 µg/mL	5 µL	10:1:4 (*v*/*v*)	vanillin- sulfuric acid	white light
	Procyanidin B2						
3	Cyanidin 3-*O*-glucoside	Anthocyanin	160 µg/mL	2 µL	5:3:1:1 (*v*/*v*)	–	white light

**Table 7:** Linearity data of the calibration curve together with LOQ and LOD values.

Standards	Linearity Range (µg/mL)	Calibration Equation ^A^	*r* ^2^	LOQ (µg/mL)	LOD (µg/mL)
Protocatechuic acid	5–50	y = 34.362x + 20.86	0.9995	0.50	0.15
Neochlorogenic acid	5–50	y = 21.462x − 2.5439	0.9992	0.09	0.03
Chlorogenic acid	5–50	y = 24.027x − 3.9582	0.9999	1.45	0.43
Procyanidin B2	5–50	y = 4.5678x − 2.239	0.9977	1.19	0.36
Epicatechin	5–50	y = 6.096x + 0.953	0.9997	1.28	0.38
Orientin	5–50	y = 25.761x + 13.279	0.999	0.49	0.15
Vitexin	5–50	y = 24.314x + 12.074	0.9973	0.64	0.19
Vitexin-2″-*O*-rhamnoside	10–100	y = 16.009x + 8.6479	0.9995	2.51	0.75
Hyperoside	5–50	y = 18.395x − 8.2302	0.9981	0.03	0.01
Rutin	10–100	y = 13.511x − 3.8909	0.9995	1.08	0.32
Isoquercitrin	5–50	y = 19.323x + 4.1729	0.9997	0.61	0.18

^A^ The calibration equation was “y = a + bx”.

**Table 8:** The closeness of the obtained results (%) by the HPLC method to their theoretical values.

Standards	Theoretical Value	Amount Found	%
Protocatechuic acid	12.5	12.23 ± 0.15	97.85
	6.25	6.02 ± 0.06	96.26
	3.125	2.78 ± 0.09	88.91
Neochlorogenic acid	12.5	12.99 ± 0.46	103.93
	6.25	7.00 ± 0.15	112.03
	3.125	3.36 ± 0.04	107.47
Chlorogenic acid	12.5	12.38 ± 0.42	99.04
	6.25	6.04 ± 0.13	96.64
	3.125	3.23 ± 0.09	103.38
Procyanidin B2	12.5	12.16 ± 0.37	97.27
	6.25	6.74 ± 0.15	107.79
	3.125	3.23 ± 0.02	103.25
Epicatechin	12.5	12.29 ± 0.28	98.28
	6.25	5.88 ± 0.18	94.13
	3.125	2.87 ± 0.03	91.78
Orientin	12.5	12.02 ± 0.24	96.18
	6.25	5.76 ± 0.14	92.22
	3.125	3.11 ± 0.15	99.48
Vitexin	12.5	12.23 ± 0.21	97.82
	6.25	5.76 ± 0.08	92.21
	3.125	2.58 ± 0.03	81.06
Vitexin-2″-*O*-rhamnoside	25	24.25 ± 0.89	97.02
	12.5	12.01 ± 0.03	96.07
	6.25	6.36 ± 0.16	101.73
Hyperoside	12.5	13.45 ± 0.23	107.56
	6.25	6.08 ± 0.08	97.36
	3.125	3.56 ± 0.18	113.94
Rutin	25	26.05 ± 0.77	104.19
	12.5	12.17 ± 0.19	97.40
	6.25	6.26 ± 0.05	100.21
Isoquercitrin	12.5	12.27 ± 0.34	98.17
	6.25	6.76 ± 0.09	108.14
	3.125	2.82 ± 0.12	90.13

Theoretical value of the standards were given as μg/mL. The average found amounts were presented as μg/mL ± SD, *n* = 3.

## Data Availability

The data presented in this study are available in this article.
